# Early versus newer generation transcatheter heart valves for transcatheter aortic valve implantation: Echocardiographic and hemodynamic evaluation of an all-comers study cohort using the dimensionless aortic regurgitation index (AR-index)

**DOI:** 10.1371/journal.pone.0217544

**Published:** 2019-05-31

**Authors:** Anja Stundl, Hannah Lucht, Jasmin Shamekhi, Marcel Weber, Alexander Sedaghat, Fritz Mellert, Eberhard Grube, Georg Nickenig, Nikos Werner, Jan-Malte Sinning

**Affiliations:** 1 Department of Medicine II, Heart Center Bonn, University Hospital Bonn, Bonn, Germany; 2 Department of Cardiothoracic Surgery, Heart Center Bonn, University Hospital Bonn, Bonn, Germany; Bern University Hospital, SWITZERLAND

## Abstract

**Aims:**

More than mild paravalvular aortic regurgitation (pAR) negatively impacts prognosis after transcatheter aortic valve implantation (TAVI). “Newer generation” transcatheter heart valves (THVs) including Direct Flow Medical, Medtronic Evolut R, Boston Lotus, and Edwards SAPIEN 3 valve system promise to improve outcome by reducing the rate of TAVI-related issues such as pAR. Aim was to evaluate and compare the hemodynamic performance with AR index of “early” vs. “newer generation” THVs and its impact on outcome.

**Methods and results:**

In 805 patients undergoing TAVI, the degree of pAR was assessed using imaging modalities (angiography, echocardiography) and hemodynamic measurements (aortic regurgitation index, ARI ratio). Severity of pAR and outcome were assessed according to the VARC-2 criteria.

805 patients underwent TAVI with use of the CoreValve (n = 400), SAPIEN XT (n = 48), Direct Flow Medical (n = 38), Evolut R (n = 114), Lotus (n = 104), or SAPIEN 3 (n = 101) prosthesis. TTE post TAVI revealed that a total of 7.3% of the patients showed moderate/severe pAR. The occurrence of greater than mild pAR occurred less frequently in patients treated with “newer generation” THVs (p<0.001): CoreValve (11.3%), SAPIEN XT (12.5%), Direct Flow Medical (5.3%), Evolut R (5.3%), Lotus (0.0%), and SAPIEN 3 (0.0%). The AR index was significantly higher (p<0.001) in patients receiving “newer generation” prostheses compared to those in whom “earlier generation” THVs were used. However, the ARI was only predictive of cumulative all-cause mortality at 1 and 3 years in “early generation”, but not in “newer generation” THVs. In the overall cohort, 30-day and 1-year mortality was 4.8% and 20.1%, respectively. In patients treated with “newer generation” devices, the respective mortality rates remained substantially below those of patients treated with “earlier generation” THVs (30-day mortality: 2.5% vs. 6.7%, p< 0.001; 1-year mortality: 11.2% vs. 27.2%, p<0.001).

**Conclusion:**

TAVI with use of “newer generation” THVs showed significantly reduced pAR and improved outcomes compared to “early generation” devices that could at least in part be explained by more favorable hemodynamics

## Introduction

Over the last decade, transcatheter aortic valve implantation (TAVI) has become an effective treatment alternative for patients suffering from severe symptomatic aortic stenosis at increased risk for surgical aortic valve replacement (SAVR) [1–3]. Among several aspects contributing to the procedural and clinical success of TAVI, appropriate patient selection, implementation of clinical best practices, and a continuous evolution in design of transcatheter heart valves (THVs) play a pivotal role to reduce the rate of TAVI-related complications, such as paravalvular aortic regurgitation (pAR). This is especially important since greater than mild pAR has been proven to have a negative impact on both short- and long-term mortality [4–5]. However, there is also evidence that mild pAR might have negative effects on survival after TAVI [5]. The heterogeneity of these data may be explained by the non-standardized clinical assessment of pAR. To address this issue, both a precise diagnosis and an accurate quantification of the severity of pAR are essential, which can be provided by a comprehensive multi-modal approach with angiographic, echocardiographic, and hemodynamic evaluation. The currently available so-called “newer generation” THVs including Direct Flow Medical, Medtronic Evolut R, Boston Lotus, and Edwards SAPIEN 3 valve system promise to facilitate the procedure itself and to further improve outcome by reducing the rate of TAVI-specific complications such as pAR due to new technology advances, which are characterized by technical improvements in design and implantation techniques (e.g. repositioning, recapturing, sealing mechanisms). Hence, the aim of the present study was to evaluate and compare the hemodynamic performance using the “AR index” and the “ARI ratio” of “early” vs. “newer generation” transcatheter heart valves and its impact on outcome.

## Methods

### Patient population

From February 2008 to September 2016, 805 consecutive patients suffering from severe, symptomatic aortic stenosis underwent TAVI with the third generation CoreValve and the Evolut R (Medtronic, Minneapolis, MN, USA), SAPIEN XT and SAPIEN 3 (Edwards Lifesciences, Irvine, CA, USA), Direct Flow Medical (Direct Flow Medical Inc., Santa Rosa, CA, USA), or Lotus (Boston Scientific, Natick, MA, USA) THV at the Heart Center Bonn, and were included into this observational retrospective study after written informed consent (**S1 Fig**).Ethics approval for the study was obtained from the local ethics committee of the University of Bonn. The TAVI procedure has been described previously [4].

The primary endpoint of this study was all-cause mortality at one year. Clinical outcomes and the severity of pAR were assessed according to the VARC-2 criteria. Follow-up data including information about the cause of death were collected during routine outpatient clinic visits, from hospital discharge letters, or via telephone interviews with the referring cardiologists or general practitioner.

### Assessment of pAR using imaging modalities

The occurrence and severity of pAR was assessed by both aortic root angiography at least ten minutes after final valve implantation in accordance with the current recommendations (position of the pigtail catheter, application of at least 25mL contrast dye at a flow rate of at least 12 mL, grading of the severity of pAR according to the Seller‘s method (according to the visually estimated density of opacification of the left ventricle) by the attending unblinded physician. In this way, pAR could be classified into four degrees: none, mild (reflow of contrast dye in the outflow tract and middle portion of the LV but clearing with each beat), moderate (reflow of contrast dye in the whole left ventricular cavity with incomplete washout in a single beat and faint opacification of the entire LV over several cardiac cycles), and severe (opacification of the entire LV with the same intensity as in the aorta and persistence of the contrast after a single beat). Apart from that, transthoracic echocardiography (TTE) was performed until day 4 after TAVI. Categorical grading of severity of pAR (0 = none-trace; 1 = mild; 2 = moderate; 3 = severe) was based on integrating available valve academic research consortium (VARC-II) criteria, including in particular circumferential extent (CE), diastolic flow reversal (DFR), regurgitation volume/regurgitation fraction, and effective regurgitant orifice area (EROA). The echocardiography for evaluation of pAR was performed by an echocardiographer who was blinded to angiographic data.

### Assessment of pAR using hemodynamic measurements

For hemodynamic evaluation of pAR, ventricular (left ventricle) and aortic (ascending aorta) pressures were simultaneously determined before and after the implant procedure by using fluid-filled catheters, and the dimensionless ARI was calculated: the gradient between aortic diastolic blood pressure (DBP) in the aorta and left ventricular end-diastolic pressure (LVEDP) was measured and adjusted for aortic systolic blood pressure (SBP) according to the formula: [(DBP—LVEDP)/SBP] x 100 [4]. To adjust the post-procedural ARI for pre-procedural status and to increase accuracy of residual pAR assessment, we also calculated the ARI ratio. „ARI ratio”was defined as post-procedural AR index in relation to pre-procedural AR index (post-procedural ARI/pre-procedural ARI) [6].

### Statistical analysis

Data are given as mean ± standard deviation if normally distributed or as median and interquartile range (IQR: quartile 1 to 3) if not normally distributed. Continuous variables were tested for normal distribution with the use of the Kolmogorov-Smirnov test. For comparison between two groups, a Student’s t-test was performed for continuous variables if normally distributed and a Mann-Whitney U test was performed for continuous variables if not normally distributed. When comparing more than two groups, ANOVA or the Kruskal-Wallis test was used. Categorical variables are given as frequencies and percentages. For categorical variables, the χ^2^-test was used for further analysis. Associations of subject characteristics and all-cause 1-year mortality were assessed by univariate und multivariate Cox proportional hazard model analyses. To limit the influence of extreme observations, clinical risk score results and labaratory values were natural log (ln)-transformed. In order to identify independent predictors of cumulative mortality, as a first step, all baseline characteristics with p≤0.05 were included in a univariate Cox proportional hazard model analysis. All variables with p≤0.05 on univariate analysis were incorporated in a stepwise multivariate Cox proportional hazard model analysis. The unadjusted cumulative event rates were estimated by Kaplan-Meier methods, and statistical assessment was performed by the log-rank test. Statistical significance was assumed when the null hypothesis could be rejected at p<0.05. Statistical analyses were conducted with SPSS Statistics Version 22.0 (IBM Corporation, Somer, NY), and MedCalc version 11.6.1.0 (MedCalc Software, Mariakerke, Belgium).

The investigators initiated the study, had full access to the data, and wrote the manuscript. All authors vouch for the accuracy and completeness of the data and all analyses and confirm that the study was conducted according to the protocol.

## Results

### Baseline characteristics

Baseline characteristics according to “early” vs. “newer generation” THVs are summarized in **Table 1.**

**Table 1:** Baseline characteristics according to “early/newer” generation THVs.

	All patients (n = 805)	Early generation THVs (n = 448)	Newer generation THVs (n = 357)	p-value
Age (years)	**80.9 ± 6.3**	81.0 ± 6.4	80.9 ± 6.2	0.886
Male gender, n (%)	**409 (50.8)**	234 (52.2)	175 (49.0)	0.365
Logistic EuroSCORE, (%)	**17.5 (11.3 to 29.8)**	20.5 (12.7 to 35.7)	14.4 (9.5 to 23.4)	**< 0.001**
EuroSCORE II, (%)	**5.3 (3.3 to 9.4)**	6.6 (3.8 to 11.4)	4.7 (3.0 to 7.7)	**<0.001**
STS-PROM, (%)	**5.2 (3.4 to 8.3)**	6.8 (4.2 to 10.7)	4.0 (2.6 to 5.7)	**< 0.001**
Body mass index, (kg/m^2^)	**26.5 ± 5.2**	26.3 ± 5.3	26.7 ± 5.0	0.291
Diabetes mellitus, n (%)	**227 (28.2)**	129 (28.8)	98 (27.5)	0.674
CAD, n (%)	**502 (62.4)**	291 (65.0)	211 (59.1)	0.089	
1-vessel- CAD, n (%)	**168 (20.9)**	99 (22.1)	69 (19.3)		
2-vessel- CAD, n (%)	**129 (16.0)**	78 (17.4)	51 (14.3)		
3-vessel- CAD, n (%)	**206 (25.6)**	114 (25.4)	92 (25.8)		
Extracardiac Arteriopathy, n (%)	**344 (42.7)**	194 (43.3)	150 (42.0)	0.714	
Atrial Fibrillation, n (%)	**338 (42.0)**	176 (39.3)	162 (45.4)	0.082	
Previous stroke, n (%)	**122 (15.2)**	75 (16.7)	47 (13.2)	0.160	
Previous MI, n (%)	**107 (13.3)**	80 (17.9)	27 (7.6)	**<0.001**	
Previous PCI, n (%)	**289 (35.9)**	167 (37.3)	122 (34.2)	0.362	
Previous cardiac surgery, n (%)	**128 (15.9)**	70 (15.6)	58 (16.2)	0.811	
COPD, n (%)	**182 (22.6)**	121 (27.0)	61 (17.1)	**0.001**	
Pulmonary hypertenion, n (%)	**288 (35.8)**	150 (33.5)	138 (38.8)	0.121	
LVEF, (%)	**52.6 ± 14.0**	49.5 ± 14.9	56.5 ± 11.7	**<0.001**	
NYHA class IV, n (%)	**100 (12.4)**	82 (18.3)	18 (5.0)	**<0.001**	
Aortic valve area, (cm^2^)	**0.71 ± 0.17**	0.70 ± 0.17	0.73 ± 0.16	**0.011**	
Pressure peak gradient, (mmHg)	**73.4 ± 25.9**	72.7 ± 26.8	74.3 ± 24.7	0.395	
Pressure mean gradient, (mmHg)	**42.0 ± 16.3**	41.7 ± 16.8	42.3 ± 15.6	0.625
CRF, n (%)	**482 (59.9)**	277 (61.8)	205 (57.4)	0.205
eGFR	**52.5 ± 18.1**	52.7 ± 19.8	52.3 ± 15.9	0.742
Dialysis, n (%)	**28 (3.5)**	13 (2.9)	15 (4.2)	0.317
NT-proBNP, (pg/mL)	**2881.0 (1098.5 to 7707.5)**	3356.0 (1191.0 to 9442.0)	2403.5 (989.8 to 5532.8)	**0.003**

A total of 805 patients with a median logistic EuroSCORE of 17.5 (IQR: 11.3 to 29.8) underwent TAVI with either the CoreValve (n = 400), SAPIEN XT (n = 48), Direct Flow Medical (n = 38), Evolut R (n = 114), Lotus (n = 104) and SAPIEN 3 (n = 101) valve system (**S1 Table**). Patients undergoing TAVI with a “early generation” THV were at higher surgical risk (Logistic EuroSCORE: 20.5 (12.7 to 35.7); EuroSCORE II: 6.6 (3.8 to 11.4); STS-PROM 6.8 (4.2 to 10.7)), and showed more comorbidities (**Table 1**) compared to patients with use of “newer generation” devices. Baseline characteristics of the study cohort (n = 805) according to ARI categories (ARI≥25 vs. ARI<25) revealed that patients with an ARI < 25 presented with significantly higher median clinical risk score results, suffered more frequently from coronary artery disease and chronic renal failure with the need of dialysis, had more often a history of previous cardiac surgery, had significantly lower eGFRs, but higher NT-proBNP levels and were clinically more likely to be in NYHA functional class IV. In short, these findings confirm that patients with an ARI < 25 appeared to be less healthy and suffer from more concomitant comorbidities (**S2 Table**).

### Periprocedural characteristics and clinical and functional outcomes

Periprocedural characteristics according to “early” vs. “newer generation” THVs and THV type are shown in **Table 2 and S3 Table**, respectively.

**Table 2:** Procedural characteristics according to “early/newer” generation THVs.

	All patients (n = 805)	Early generation THVs (n = 448)	Newer generation THVs (n = 357)	p-value
Access site				**0.001**
Trans- femoral, n(%)	785 (97.5)	428 (95.5)	357 (100.0)	
Trans- subclavian, n (%)	14 (1.7)	14 (3.1)	0 (0.0)	
Trans-aortic, n (%)	6 (0.6)	6 (1.3)	0 (0.0)	
Prosthesis size				**<0.001**
20 mm, n(%)	1 (0.1)	1 (0.2)	0 (0.0)	
23 mm, n(%)	96 (11.9)	32 (7.1)	64 (17.9)	
25 mm, n(%)	49 (6.1)	0 (0.0)	49 (13.7)	
26 mm, n(%)	230 (28.6)	161 (35.9)	69 (19.3)	
27 mm, n(%)	54 (6.7)	0 (0.0)	54 (15.1)	
29 mm, n(%)	308 (38.3)	187 (41.7)	121 (33.9)	
31 mm, n(%)	67 (8.3)	67 (15.0)	0 (0.0)	
Annulus diameter, (mm)	23.9 ± 2.4	23.6 ± 2.4	24.2 ± 2.4	**<0.001**
Maximum diameter, (mm)	26.8 ± 2.8	26.3 ± 2.7	27.5 ± 2.7	**<0.001**
Minimum diameter, (mm)	21.0 ± 2.4	20.6 ± 2.1	21.5 ± 2.6	**<0.001**
Pre-procedural aortic regurgitation index (AR index)	34.2 ± 10.5	33.3 ± 10.5	35.1 ± 10.5	**0.023**
Pre-dilation, n (%)	408 (50.7)	256 (57.1)	152 (42.7)	**<0.001**
Post-dilation, n (%)	200 (24.8)	155 (34.6)	45 (12.6)	**<0.001**
Procedure time, (min.)	63.0 (50.0 to 86.0)	66.0 (50.0 to 88.5)	60.0 (48.8 to 83.0)	**0.019**

The majority of the patients (97.5%) underwent TAVI via transfemoral approach. Patients who received “newer generation” THVs, in particular with use of either the Direct Flow Medical or the Lotus valve system already showed more beneficial pre-implant hemodynamics as assessed by the pre-procedural ARI (Direct Flow Medical: 36.2 ± 10.7; Lotus: 36.5 ± 11.2) compared to those with use of another prosthesis type (CoreValve: 33.3 ± 10.6; SAPIEN XT: 33.2 ± 9.9; Evolut R: 32.8 ± 10.7; and SAPIEN 3: 35.9 ± 8.9, p = 0.020).

Clinical and functional outcomes according to to “early” vs. “newer generation” THVs and the THV type are summarized in **Table 3** and **S4 Table**, respectively.

**Table 3:** Clinical and functional outcomes according to “early/newer” generation THVs.

	All patients (n = 805)	Early generation THVs (n = 448)	Newer generation THVs (n = 357)	p-value
30-day mortality, n(%)	39 (4.8)	30 (6.7)	9 (2.5)	**0.006**
180-day mortality, n(%)	116 (14.4)	85 (19.0)	31 (8.7)	**<0.001**
1-year mortality, n(%)	162 (20.1)	122 (27.2)	40 (11.2)	**<0.001**
2-year mortality, n(%)	211 (26.2)	162 (36.2)	49 (13.7)	**<0.001**
3-year mortality, n(%)	245 (30.4)	193 (43.1)	52 (14.6)	**<0.001**
Stroke, n (%)	20 (2.5)	14 (3.1)	6 (1.7)	0.191
Myocardial infarction, n(%)	6 (0.7)	5 (1.1)	1 (0.3)	0.171
Minor vascular complications, n(%)	154 (19.1)	96 (21.4)	58 (16.2)	0.063
Major vascular complications, n(%)	32 (4.0)	27 (6.0)	5 (1.4)	**0.001**
Major bleedings, n(%)	36 (4.5)	33 (7.4)	3 (0.8)	**<0.001**
Pacemaker implantation, n(%)	124 (15.4)	75 (16.7)	49 (13.7)	0.498
Acute kidney injury, n(%)	125 (15.5)	93 (20.8)	32 (9.0)	**<0.001**
Angiographic data				**<0.001**
None pAR, n (%)	342 (42.5)	126 (28.1)	216 (60.5)	
Mild pAR, n (%)	403 (50.1)	270 (60.3)	133 (37.3)	
Moderate pAR, n (%)	55 (6.8)	47 (10.5)	8 (2.2)	
Severe pAR, n (%)	5 (0.6)	5 (1.1)	0 (0.0)	
More than mild pAR, n(%)	59 (7.3)	51 (11.4)	8 (2.2)	**<0.001**
Post-procedural aortic regurgitation index (AR index, ARI)	29.2 ± 8.4	28.0 ± 8.2	30.7 ± 8.5	**<0.001**
AR index < 25, n(%)	235 (29.2)	147 (32.8)	88 (24.6)	**0.011**
ARI ratio	0.92 ± 0.43	0.92 ± 0.51	0.93 ± 0.33	0.804

**Fig 1:**
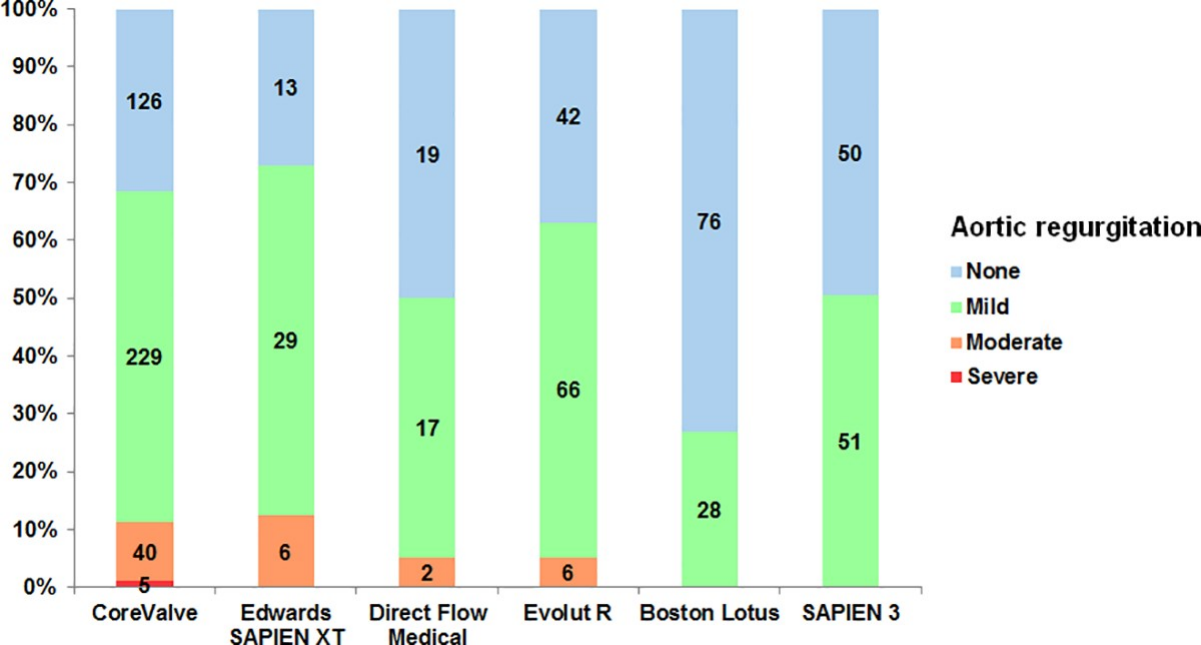
Distribution of pAR severity according to THV type. The bar diagram shows that 326/805 (40.5%) of the patients had no relevant pAR, 420/805 (52.2%) trace or mild pAR, whereas 59/805 (7.3%) of the patients suffered from moderate/severe pAR in TTE post TAVI.

30-day and 1-year overall all-cause mortality were 4.8% (39/805) and 20.1% (171/805), respectively. TTE post TAVI revealed that 326/805 (40.5%) of the patients had no relevant pAR, 420/805 (52.2%) trace or mild pAR, whereas 59/805 (7.3%) of the patients suffered from moderate/severe pAR (**Fig 1**).

A clear correlation between severity of pAR and AR index was found (**S5 Table**). Greater than mild pAR occurred significantly less frequently in patients undergoing TAVI with “newer generation” prostheses (p<0.001): CoreValve (11.3%), SAPIEN XT (12.5%), Direct Flow Medical (5.3%), Evolut R (5.3%), Lotus (0.0%), and SAPIEN 3 (0.0%).

**Fig 2:**
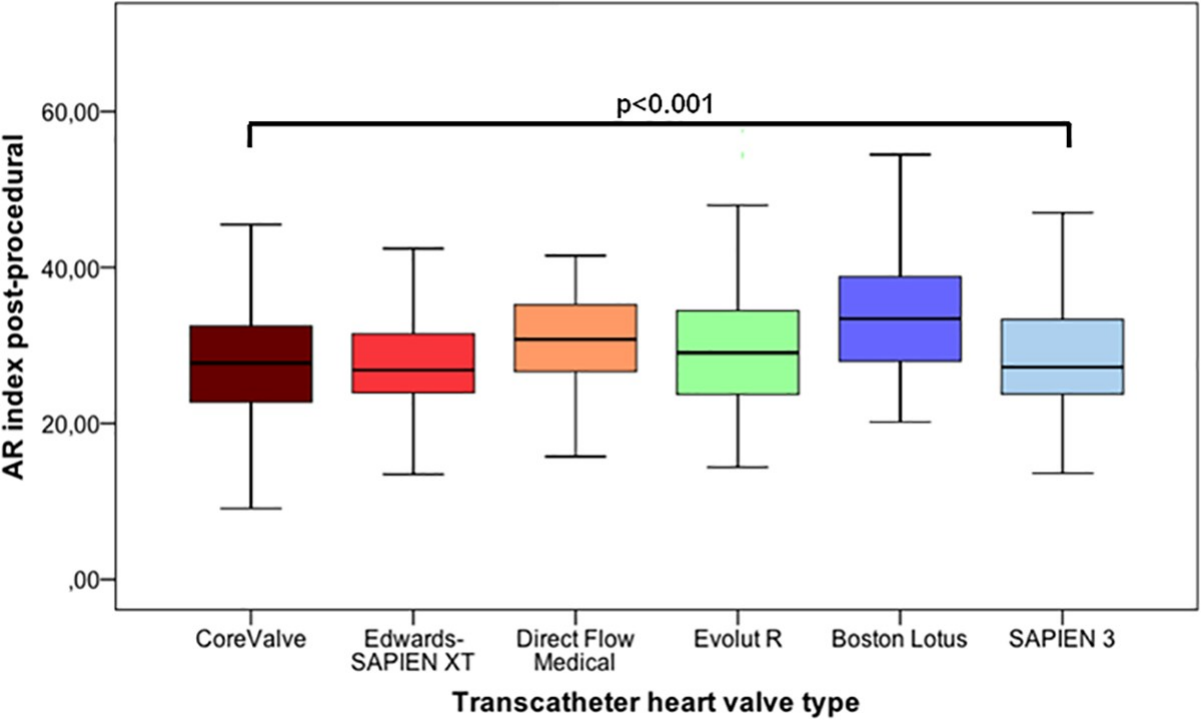
Hemodynamic evaluation of THVs using the aortic regurgitation index. Hemodynamic evaluation of THVs using the ARI. The post-procedural ARI was significantly higher (p<0.001) with use of the Lotus (34.2 ± 8.7), and the Direct Flow Medical (30.8 ± 6.9) THV compared with the CoreValve (28.1 ± 8.3), SAPIEN XT (27.7 ± 7.2), Evolut R (29.6 ± 9.2), and SAPIEN 3 (28.4 ± 6.9) prosthesis.

**Fig 3:**
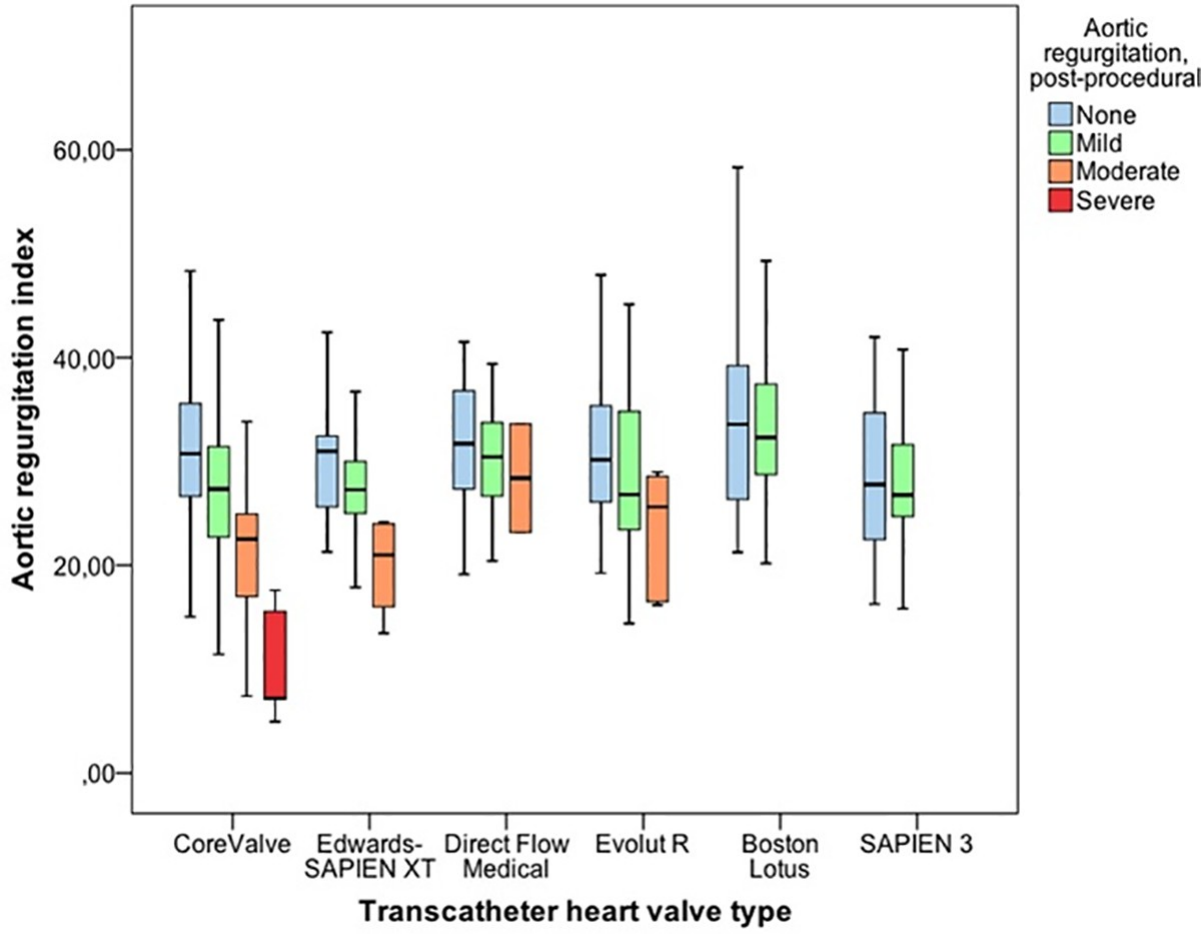
Post-procedural aortic regurgitation index stratified by degree of pAR and THV type. Greater than mild pAR occurred significantly less frequently in patients undergoing TAVI with “newer generation” THVs and the post-procedural ARI was significantly higher with use of the Lotus and the Direct Flow Medical THV compared to all other THVs.

The post-procedural ARI was significantly higher (p<0.001) with use of “newer generation” THVs (30.7 ± 8.5) compared to „early generation”THVs (28.0 ± 8.2) (data not shown). The Lotus (34.2 ± 8.7) and Direct Flow Medical (30.8 ± 6.9) prostheses have been the best performing valve systems in direct comparison to CoreValve (28.1 ± 8.3), SAPIEN XT (27.7 ±7.2), Evolut R 29.6 ± 9.2) and SAPIEN 3 (28.4 ± 6.9) THV (**Figs 2 and 3**).

**Fig 4:**
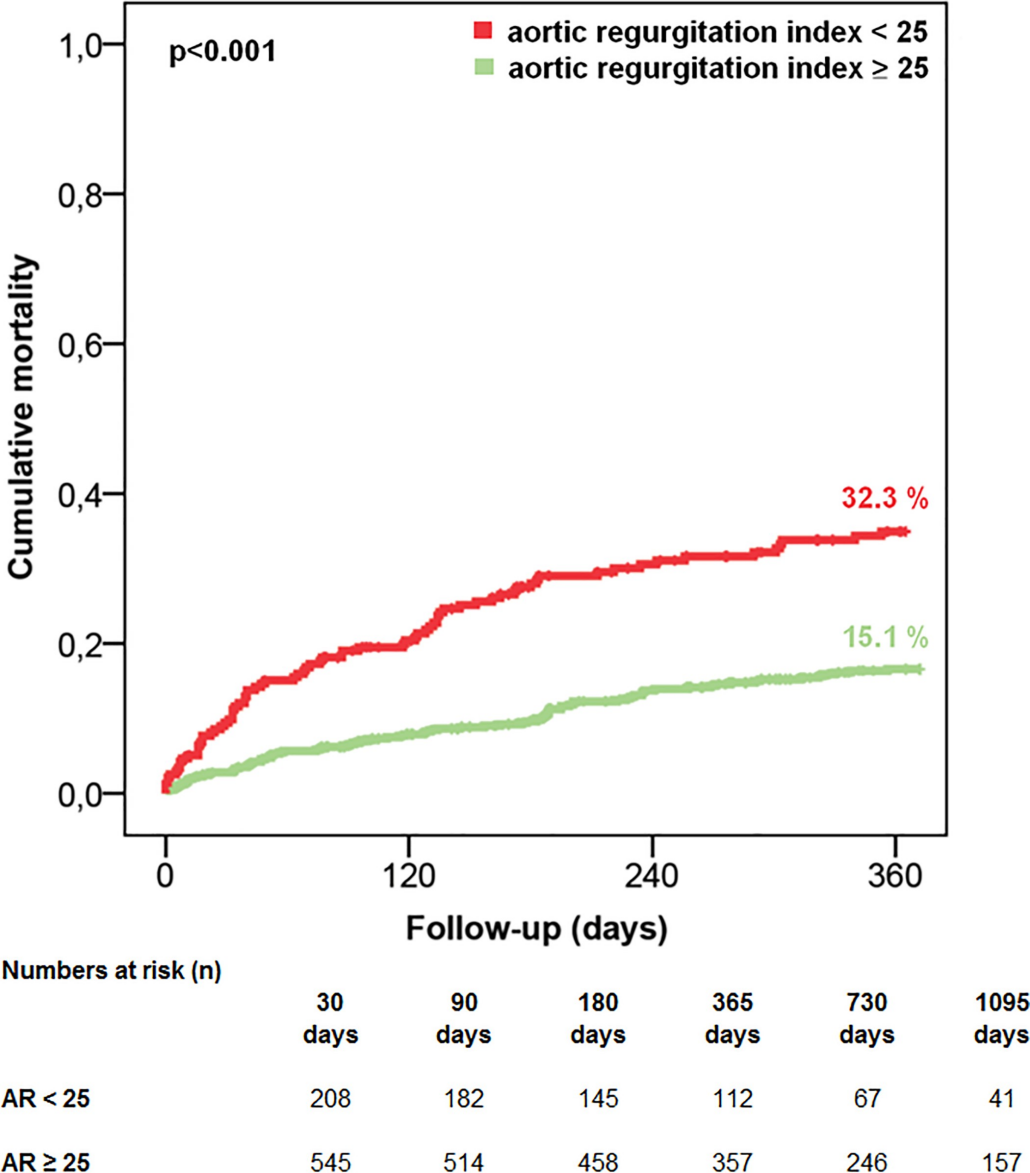
All-cause 1-year mortality according to AR index. A post-procedural ARI less than 25 was associated with significantly increased all-cause mortality at 1 year (32.3% vs. 15.1%, p<0.001).

A post-procedural ARI less than 25 was associated with significantly increased all-cause mortality at 1 year and 3 years (1 year: 32.3% vs. 15.1%, p<0.001; 3 years: 43.8% vs. 24.9%, p<0.001) **(Fig 4, S2 Fig)**.

**Fig 5:**
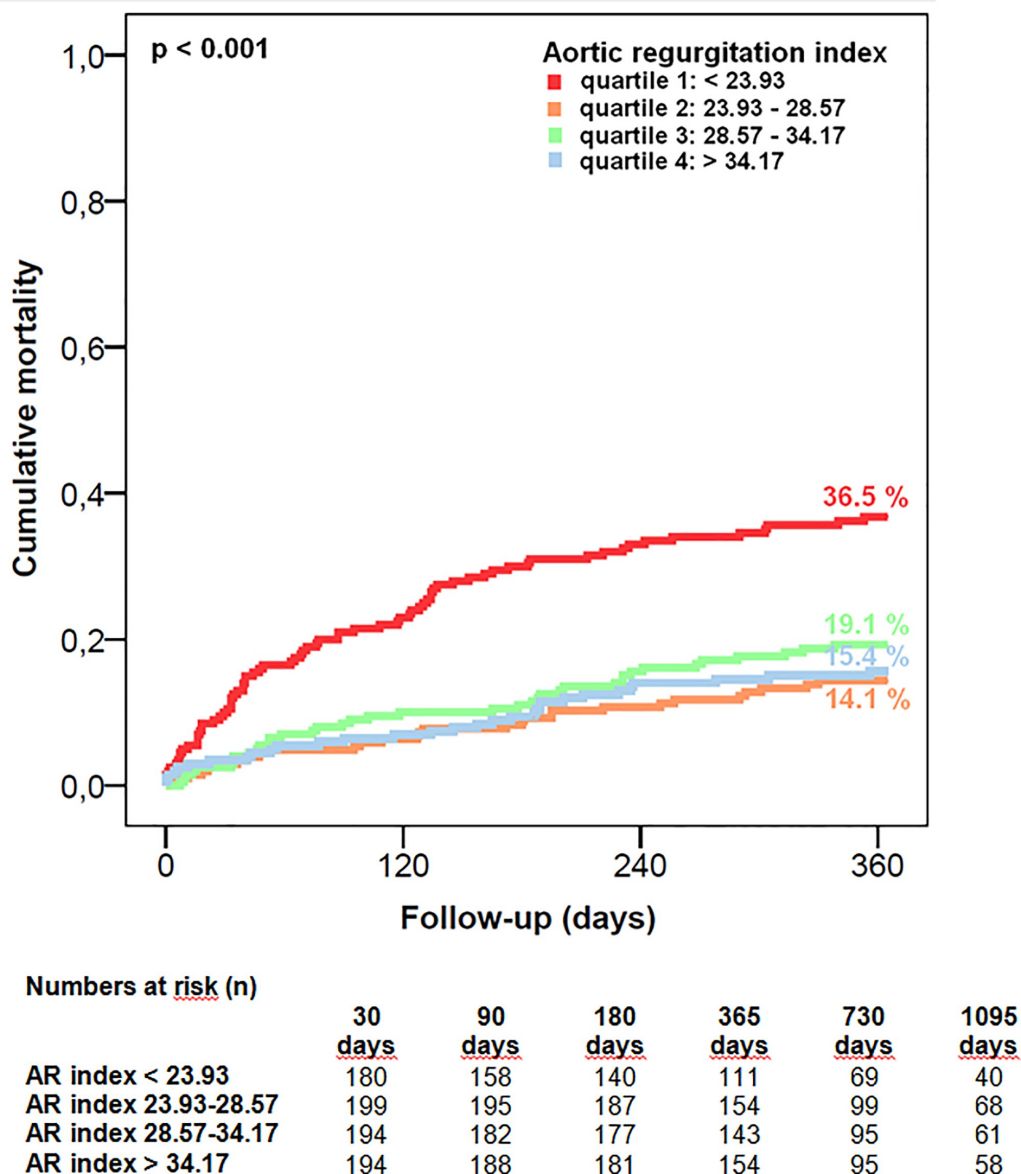
All-cause 1-year mortality according to aortic regurgitation index quartiles. When categorized into quartiles, a post-procedural ARI in the lowermost quartile (<23.93) was significantly associated with increased all-cause mortality at 1 year (Q1: < 23.93: 36.5%, Q2: 23.93–28.57: 14.1%, Q3: 28.57–34.17: 19.1%, Q4: > 34.17: 15.4%; p<0.001).

To point out the differences in mortality, however, a categorization into quartiles was performed by which comparable results could be obtained; a post-procedural ARI in the lowermost quartile (<23.93) displayed a significant association with mortality at 365 and 1095 days (Q1: < 23.93: 36.5% and 51.0%, Q2: 23.93–28.57: 14.1% and 30.7%, Q3: 28.57–34.17: 19.1% and 29.6%, Q4: > 34.17: 15.4% and 23.9%; p<0.001) (**Fig 5, S3 Fig**).

With a cut-off value of 0.60, which has been recently shown to be the optimal cut-off for the prediction of 1-year mortality in patients with an ARI < 25 [6], the patients were similarly categorized into groups as follows: ARI_post_ < 25 with ARI ratio < 0.60, ARI_post_ < 25 with ARI ratio ≥ 0.60, and ARI_post_ ≥ 25. TAVI patients with ARI_post_ < 25 and ARI ratio < 0.60 were characterized by a significantly higher 1-year and 3-year mortality rate (40.6% and 53.6%) compared to those with ARI_post_ < 25 and ARI ratio ≥ 0.60 (28.7% and 43.3%) or ARI_post_ ≥ 25 (16.1% and 28.1%), respectively (p<0.001) (**S4 Fig**).

TAVI-related complications such as major vascular complications, and major bleeding were less frequent in patients who received “newer generation” THVs, whereas the rate of pacemaker implantation due to new-onset conduction disturbances was particularly high in patients who underwent TAVI with use of the Lotus valve system (Lotus 23.1%; CoreValve: 18.3%; SAPIEN XT: 4.2%; Direct Flow Medical: 7.9%; Evolut R: 14.0%; SAPIEN 3: 5.9%; p = 0.009).

### Predictors of cumulative all-cause 1-year and 3-year mortality

Based on Table 1, univariate cox proportional hazard model analysis revealed that–when stratified according to “early generation” vs. “newer generation” THVs—logistic EuroSCORE, EuroSCORE II, STS-PROM, left ventricular ejection fraction, NYHA functional class IV, maximum annulus diameter in CT scan, the need for post-dilation, major vascular complications, major bleedings, acute kidney injury, more than mild pAR, an ARI < 25 and post-procedural aortic regurgitation were associated with an increased risk for cumulative 1-year mortality for “early generation” devices, whereas for “newer generation” prostheses, however, the same analysis showed that—apart from the existing risk scores—COPD, NYHA functional class IV, access site, major vascular complications, major bleedings, and acute kidney injury were attributed to an increased risk for cumulative 1-year mortality. In a supplementary multivariate cox proportional hazard model analysis, the logistic EuroSCORE, acute kidney injury and ARI less than 25 remained independent predictors for “early generation” THVs, whereas, for “newer generation” THVs, COPD, NYHA functional class IV, access site, major bleedings, and acute kidney injury appeared to be independently associated with an increased risk for cumulative 1-year mortality. For cumulative 3-year mortality, however, univariate cox proportional hazard model analysis revealed that–when stratified according to “early generation” vs. “newer generation” THVs—logistic EuroSCORE, EuroSCORE II, STS-PROM, previous myocardial infarction, left ventricular ejection fraction, NYHA functional class IV, NT-proBNP, maximum annulus diameter in CT scan, the need for post-dilation, major vascular complications, major bleedings, acute kidney injury, more than mild pAR, an ARI < 25 and post-procedural aortic regurgitation were associated with an increased risk for “early generation” devices. In an additional multivariate cox proportional hazard model analysis, the logistic EuroSCORE, NT-proBNP, maximum annulus diameter in CT scan, acute kidney injury and an AR less than 25 remained independent predictors. When stratified according to “newer generation” THVs, however, univariate cox proportional hazard model analysis revealed that logistic EuroSCORE, EuroSCORE II, STS-PROM, NYHA functional class IV, access site, major vascular complications, major bleedings, and acute kidney injury, were associated with an increased risk for cumulative 3-year mortality. After application of a multivariate cox proportional hazard model, the EuroSCORE II, access site, major bleeding and acute kidney injury remained independent predictors for cumulative 3-year mortality (**Tables 4 and 5**).

**Table 4:** Univariate and multivariate cox proportional hazard model analysis (prediction of 1-year mortality) stratified according to “early generation” vs. “newer generation” transcatheter heart valves–hazard ratios with 95% CI.

Early generation THVs	Univariate HR (95% CI)	p-value	Multivariate HR (95% CI)	p-value	Newer generation THVS	Univariate HR (95% CI)	p-value	Multivariate HR (95% CI)	p-value
Logistic EuroSCORE^1^	1.610 (1.379–1.880)	**<0.001**	1.420 (1.049–1.922)	**0.023**	Logistic EuroSCORE^1^	1.366 (1.071–1.742)	**0.012**		
EuroSCORE II^1^	1.458 (1.261–1.686)	**<0.001**			EuroSCORE II^1^	1.364 (1.108–1.680)	**0.003**		
STS-PROM^1^	1.444 (1.265–1.649)	**<0.001**			STS-PROM^1^	1.426 (1.219–1.669)	**<0.001**		
Previous MI	1.403 (0.915–2.152)	0.120			Previous MI	1.375 (0.489–3.864)	0.546		
COPD	1.448 (0.995–2.109)	0.053			COPD	2.342 (1.191–4.608)	**0.014**	2.022 (1.010–4.045)	**0.047**
LVEF^1^	0.671 (0.565–0.796)	**<0.001**			LVEF^1^	0.992 (0.726–1.355)	0.960		
NYHA IV	2.227 (1.514–3.278)	**<0.001**			NYHA IV	4.503 (1.991–10.182)	**<0.001**	3.093 (1.197–7.993)	**0.020**
AVA^1^	1.083 (0.905–1.296)	0.383			AVA^1^	0.970 (0.712–1.320)	0.845		
NT-proBNP^1^	1.102 (0.997–1.219)	0.058			NT-proBNP^1^	1.101 (0.893–1.356)	0.369		
Access site	0.819 (0.515–1.302)	0.398			Access site	3.710 (2.239–6.149)	**<0.001**	3.014 (1.673–5.429)	**<0.001**
Prosthesis size	1.049 (0.968–1.137)	0.243			Prosthesis size	1.132 (0.975–1.313)	0.103		
Annulus diameter^1^	1.191 (0.991–1.430)	0.062			Annulus diameter^1^	1.212 (0.879–1.673)	0.241		
Maximum diameter^1^	1.342 (1.034–1.742)	**0.027**	1.404 (0.986–1.999)	0.060	Maximum diameter^1^	1.372 (0.875–2.152)	0.168		
Minimum diameter^1^	1.202 (0.937–1.543)	0.148			Minimum diameter^1^	1.105 (0.693–1.760)	0.675		
Pre-procedural AR index^1^	1.072 (0.881–1.304)	0.490			Pre-procedural AR index^1^	0.826 (0.597–1.143)	0.249		
Pre-dilation	1.130 (0.787–1.623)	0.507			Pre-dilation	1.078 (0.574–2.027)	0.815		
Post-dilation	1.499 (1.047–2.146)	**0.027**			Post-dilation	1.528 (0.676–3.455)	0.309		
Procedure time^1^	1.049 (0.878–1.254)	0.596			Procedure time^1^	1.058 (0.787–1.423)	0.707		
Major vascular complications	2.546 (1.458–4.445)	**0.001**			Major vascular complications	5.643 (1.360–23.413)	**0.017**		
Major bleedings	2.196 (1.279–3.771)	**0.004**			Major bleedings	11.802 (2.839–49.058)	**0.001**	10.608 (2.414–46.605)	**0.002**
Acute kidney injury	2.556 (1.762–3.708)	**<0.001**	2.756 (1.326–5.727)	**0.007**	Acute kidney injury	4.884 (2.371–10.062	**<0.001**	4.375 (2.068–9.256)	**<0.001**
More than mild pAR	2.889 (1.872–4.460)	**<0.001**			More than mild pAR	1.006 (0.138–7.321)	0.996		
ARI < 25	2.679 (1.877–3.823)	**<0.001**	3.134 (1.585–6.199)	**0.001**	ARI < 25	1.607 (0.829–3.116)	0.160		
Post-procedural AR index^1^		**<0.001**			Post-procedural AR index^1^	0.757 (0.541–1.060)	0.105		

^1^ per 1-SD increase

ARI = aortic regurgitation index; AVA = aortic valve area; CI = confidence interval; COPD = chronic obstructive pulmonary disease; EuroSCORE = European System for Cardiac Operative Risk Evaluation; HR = hazard ratio; LVEF = left ventricular ejection fraction; MI = myocardial infarction; NT-proBNP = N-terminal pro-brain natriuretic peptide; NYHA = New York Heart Association; STS = Society of Thoracic Surgery.

**Table 5:** Univariate and multivariate cox proportional hazard model analysis (prediction of 3-year mortality) stratified according to “early generation” vs. “newer generation” transcatheter heart valves–hazard ratios with 95% CI.

Early generation THVs	Univariate HR (95% CI)	p-value	Multivariate HR (95% CI)	p-value	Newer generation THVs	Univariate HR (95% CI)	p-value	Multivariate HR (95% CI)	p-value
Logistic EuroSCORE^1^	1.503 (1.324–1.706)	**<0.001**	1.490 (1.147–1.936)	**0.003**	Logistic EuroSCORE^1^	1.389 (1.128–1.711)	**0.002**		
EuroSCORE II^1^	1.376 (1.201–1.577)	**<0.001**			EuroSCORE II^1^	1.362 (1.127–1.645	**0.001**	1.301 (1.070–1.582)	**0.008**
STS-PROM^1^	1.438 (1.286–1.607)	**<0.001**			STS-PROM^1^	1.437 (1.239–1.666)	**<0.001**		
Previous MI	1.492 (1.062–2.094)	**0.021**			Previous MI	1.830 (0.820–4.084)	0.140		
COPD	1.474 (1.090–1.992)	**0.012**			COPD	1.790 (0.954–3.356)	0.070		
LVEF^1^	0.786 (0.683–0.904))	**0.001**	1.333 (0.979–1.814)	0.068	LVEF^1^	0.836 (0.643–1.086)	0.180		
NYHA IV	1.857 (1.338–2.575)	**<0.001**			NYHA IV	3.150 (1.414–7.019)	**0.005**		
AVA^1^	1.045 (0.906–1.207)	0.545			AVA^1^	0.853 (0.646–1.128)	0.265		
NT-proBNP^1^	1.148 (1.069–1.234)	**<0.001**	1.807 (1.295–2.522)	**0.001**	NT-proBNP^1^	1.134 (0.956–1.344)	0.149		
Access site	0.746 (0.490–1.136)	0.172			Access site	3.710 (2.239–6.149)	**<0.001**	3.191 (1.865–5.462)	**<0.001**
Prosthesis size	1.022 (0.959–1.088)	0.502			Prosthesis size	1.068 (0.940–1.214)	0.311		
Annulus diameter^1^	1.130 (0.975–1.308)	0.104			Annulus diameter^1^	1.139 (0.856–1.514)	0.372		
Maximum diameter^1^	1.242 (1.015–1.520)	**0.036**	1.709 (1.223–2.387)	**0.002**	Maximum diameter^1^	1.446 (0.943–2.217)	0.091		
Minimum diameter^1^	1.128 (0.930–1.368)	0.221			Minimum diameter^1^	1.096 (0.715–1.681)	0.674		
Pre-procedural AR index^1^	0.986 (0.842–1.154)	0.857			Pre-procedural AR index^1^	0.874 (0.656–1.164)	0.356		
Pre-dilation	1.036 (0.778–1.380)	0.809			Pre-dilation	1.276 (0.726–2.243)	0.397		
Post-dilation	1.365 (1.022–1.823)	**0.035**			Post-dilation	1.576 (0.736–3.373)	0.242		
Procedure time^1^	0.971 (0.832–1.134)	0.712			Procedure time^1^	1.063 (0.816–1.384)	0.650		
Major vascular complications	2.186 (1.345–3.553)	**0.002**			Major vascular complications	4.487 (1.088–18.501)	**0.038**		
Major bleedings	2.051 (1.303–3.228)	**0.002**			Major bleedings	10.858 (2.616–45.065)	**0.001**	11.309 (2.675–47.810)	**0.001**
Acute kidney injury	2.153 (1.578–2.936)	**<0.001**	2.209 (1.227–3.977)	**0.008**	Acute kidney injury	3.800 (1.924–7.509)	**<0.001**	4.045 (2.027–8.070)	**<0.001**
More than mild pAR	2.166 (1.466–3.200)	**<0.001**	0.291 (0.069–1.235)	0.094	More than mild pAR	0.819 (0.113–5.931)	0.843		
ARI < 25	2.289 (1.721–3.044)	**<0.001**	2.561 (1.477–4.441)	**0.001**	ARI < 25	1.485 (0.811–2.716)	0.200		
Post-procedural AR index^1^	0.637 (0.542–0.750)	**<0.001**			Post-procedural AR index^1^	0.794 (0.591–1.066)	0.125		

^1^ per 1-SD increase

ARI = aortic regurgitation index; AVA = aortic valve area; CI = confidence interval; COPD = chronic obstructive pulmonary disease; EuroSCORE = European System for Cardiac Operative Risk Evaluation; HR = hazard ratio; LVEF = left ventricular ejection fraction; MI = myocardial infarction; NT-proBNP = N-terminal pro-brain natriuretic peptide; NYHA = New York Heart Association; STS = Society of Thoracic Surgery.

## Discussion

In this study, we were able to show that, among the several aspects contributing to the improvement of procedural and clinical outcome of TAVI patients such as more appropriate patient selection with decreasing surgical risk, implementation of best clinical practices, and growing experience (the so-called "learning curve"), the use of “newer generation” THVs significantly reduces the occurrence of pAR and, thus, helps to further improve patient outcomes. Greater than mild pAR occurred significantly less frequently in patients undergoing TAVI with “newer generation” THVs and the post-procedural ARI was significantly higher with use of the Lotus THV compared to all other THVs. When categorized into quartiles, a post-procedural ARI in the lowermost quartile was significantly associated with increased all-cause mortality at 1 and 3 years. However, an ARI lower than 25 was only predictive for 1-year and 3-year mortality in “early generation”, but not in “newer generation” THVs, since the occurrence of more than mild pAR was significantly lower with these valve types. Taken together, this improvement in procedural success and patient outcome might also be explained to a certain extent by more favorable hemodynamics as assessed with the ARI and ARI ratio compared to “early generation” THVs.

Meanwhile, TAVI has grown rapidly to a widely accepted procedure with more than >200.000 AS patients implanted worldwide and a continued positive trend. Recently, results from the randomized Placement of Aortic Transcatheter Valves (PARTNER) 2 trial demonstrated that, although the incidence of residual pAR was higher in the TAVI group, the overall hemodynamic improvement appeared to be excellent reflected in lower mean gradients, lower incidence in moderate-to-severe prosthesis-patient-mismatch, and significantly greater mean aortic valve areas at 1 year [2]. The 1-year results of the SAPIEN 3 observational study were even superior to those from PARTNER 2 underlining the advantages of a “newer generation” THV [7]. These results were confirmed by the CoreValve Evolut R FORWARD registry study demonstrating low mortality and excellent hemodynamics in patients undergoing TAVI with use of a “newer generation device” [8].

Consistent with these previous data, we were able to show that 1-year mortality ranged between 28.5% at its highest with the CoreValve prosthesis and 9.6% with the Lotus valve system. The significant mortality reduction with “newer generation” THVs might also be explained, among the aforementioned aspects related to the procedural and clinical success of TAVI (appropriate patient selection, implementation of a set of clinical best practices, TAVI learning curve) by special design features. In spite of the improved outcome, however, pAR caused by sub-optimal expansion of the prosthesis frame and incomplete circumferential apposition at the level of the aortic annulus [9–13] remains a TAVI-specific phenomenon following TAVI that has been associated with increased mortality [4–5,14–15]. The incidence of greater than mild pAR reported in “early generation” THV studies is thought to be between 4% to 15% [4,16–18]. With the advent of “newer generation” THVs, satisfactory results in terms of pAR could have been achieved. In the CoreValve Evolut R CE Study, relevant pAR after TAVI was documented in 3.4% of the cases presumably being attributed to optimized oversizing, consistent radial force across the annulus diameter and an extended sealing skirt [19]. As shown in the REPRISE II study, the Lotus valve system is also characterized by very low rates of pAR; in only 1.7% of the patients greater than mild pAR could be detected. It incorporates the adaptive seal technique to minimize pAR and is characterized by complete repositionability and retrievability [20–21]. Likewise, less than two percent of the TAVI patients in the SAPIEN 3 observational study developed greater than mild pAR which might be due an outer skirt at the distal part of the valve and its fine positioning control [22]. This coincides with our finding that the introduction of “newer generation” THVs led to a considerable reduction in the occurrence of relevant, greater than mild pAR compared to “early generation” THVs.

In the previous studies mentioned above [7,19], hemodynamic performance was assessed using echocardiographic parameters such as aortic valve pressure gradients and mean aortic valve areas. In contrast, we used a multimodal approach combining imaging modalities (angiography, echocardiography) and hemodynamic measurements such as the ARI and/or the ARI ratio which might be useful to more precisely assess the severity of pAR. In addition to echocardiographic evaluation of hemodynamic performance by recording the gradient and degree of pAR, the dimensionless ARI represents a useful hemodynamic parameter to accurately quantify the degree of pAR immediately after the procedure and to distinguish between relevant (greater than mild) and non-relevant pAR [4,23–24]. Despite the commonly known limitations (confounding of the ARI due to heart rate, concomitant diastolic dysfunction, etc.) [24], a cut-off value of 25 has been shown to have a high negative predictive value for the occurrence of greater than mild pAR and to independently predict 1-year mortality following TAVI [4]. With regard to this last point, we were able to confirm in the present study, that an ARI below the cut-off value of 25 was independently associated with an increased risk of cumulative all-cause 1-year and 3-year mortality, albeit only in patients treated with “early generation” THVs. We consider this observation to be plausible as with lower rates of pAR in newer-generation valve patients, the attributable fraction of patients dying due to relevant, more than mild pAR decreases. Furthermore, it seems to be obvious that the specificity of ARI in detecting relevant pAR possibly declines if the prevalence of post-procedural pAR decreases.

Given its good predictive power, the ARI is regularly applied in our clinical daily routine and serves as an indicator whether corrective measures should be applied or not. Considering the potential pitfalls of determination of the ARI alone, we were recently able to show, that it would be also appropriate to further take into account the pre-procedural hemodynamic status in the form of the ARI ratio (ARI_post_/ARI_pre_). A value of 0.60 was identified to be the optimal cut-off for the prediction of 1-year mortality after TAVI. Depending on the value, a treatment algorithm was developed to provide better assistance in identifying patients with relevant pAR who might benefit from corrective measures [6]. This fact could be also confirmed in the present study, and both ARI and ARI ratio turned out to be helpful tools for assessing hemodynamic performance and grading pAR further on. In patients undergoing TAVI with the use of “newer generation” THVs, especially in patients who received the Lotus valve system, hemodynamic performance using the ARI and ARI ratio was more favorable than in patients who received “early generation” THVs. The positive hemodynamic result might be due to an ever-growing operator’s experience but with a much greater extent due to the new devices and their design features, and might be one explanation for the positive trend towards increased survival after TAVI.

### Study limitations

This study reflects clinical daily routine. Therefore, study results are confounded by the shift to less sick patients. In addition, a certain learning curve effect with increase of patient numbers and growing experience and the implementation of clinical best practices has to be emphasized. Therefore, we cannot exclude that potential selection and/or therapy bias and unmeasured confounders may have affected our results. Besides, the single-center character is a further limitation of the study. For further verification and generalization of our results, larger studies are needed.

## Conclusions

TAVI with use of “newer generation” THVs significantly reduced pAR and improved outcomes compared to “early generation” devices that might at least in part be explained by more beneficial hemodynamics.

## Supporting information

S1 Fig Study flow chart. Flow chart showing the included patients and the distribution of THVs.
(TIF)

S2 Fig All-cause 3-year mortality according to AR index. A post-procedural ARI less than 25 was associated with significantly increased all-cause mortality at 3 years (43.8% vs. 24.9%, p<0.001).
(TIF)

S3 Fig All-cause 3-year mortality according to aortic regurgitation index quartiles. When categorized into quartiles, a post-procedural ARI in the lowermost quartile (<23.93) was significantly associated with increased all-cause mortality at 3 years (Q1: < 23.93: 51.0%, Q2: 23.93–28.57: 30.7%, Q3: 28.57–34.17: 29.6%, Q4: > 34.17: 23.9%; p<0.001).
(TIF)

S4 Fig All-cause 3-year mortality according to post-procedural aortic regurgitation index and ARI ratio. In the entire cohort, TAVI patients with ARI_post_ < 25 and ARI ratio < 0.60 showed a significant higher 3-year mortality rate (53.6%) compared to those with ARI_post_ < 25 and ARI ratio ≥ 0.60 (43.3%) or ARI_post_ ≥ 25 (28.1%), respectively (p<0.001).
(TIF)

S1 Table Baseline characteristics according to transcatheter heart valve type. (DOCX)

S2 Table Baseline characteristics according to AR index. (DOCX)

S3 Table Procedural characteristics according to the transcatheter heart valve type. (DOCX)

S4 Table Clinical and functional outcomes according to the transcatheter heart valve type. (DOCX)

S5 Table Severity of pAR and AR index. (DOCX)

## Abbreviations

(p)AR: (Paravalvular) aortic regurgitation
ARI: Aortic regurgitation index
EuroSCORE: European System for Cardiac Operative Risk Evaluation
LVEF: Left ventricular ejection fraction
NYHA: New York Heart Association
SAVR: Surgical aortic valve replacement
STS-PROM: The Society of Thoracic Surgeons' Predicted Risk of Mortality score
TAVI: Transcatheter aortic valve implantation
THV: Transcatheter heart valve
VARC: Valve Academic Research Consortium
