# Supplementary figures and images for: Early versus newer generation transcatheter heart valves for transcatheter aortic valve implantation: Echocardiographic and hemodynamic evaluation of an all-comers study cohort using the dimensionless aortic regurgitation index (AR-index)
Source: PLoS One. 2019 May 31;14(5):e0217544. doi: 10.1371/journal.pone.0217544 (PMC6544262; doi:10.1371/journal.pone.0217544)

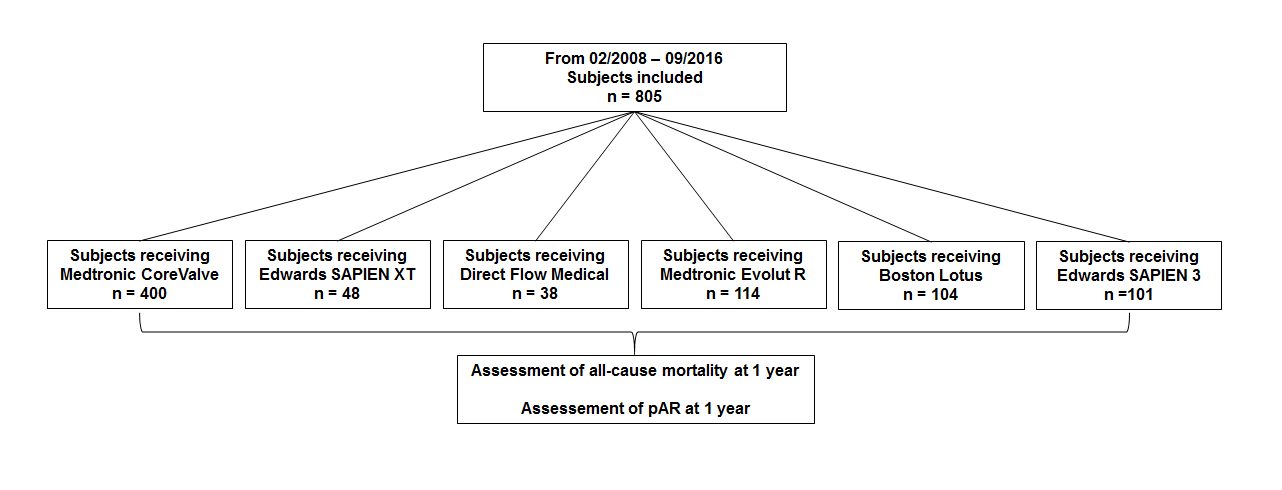

Supplement: S1 Fig — Flow chart showing the included patients and the distribution of THVs. (TIF) [file pone.0217544.s001.tif]

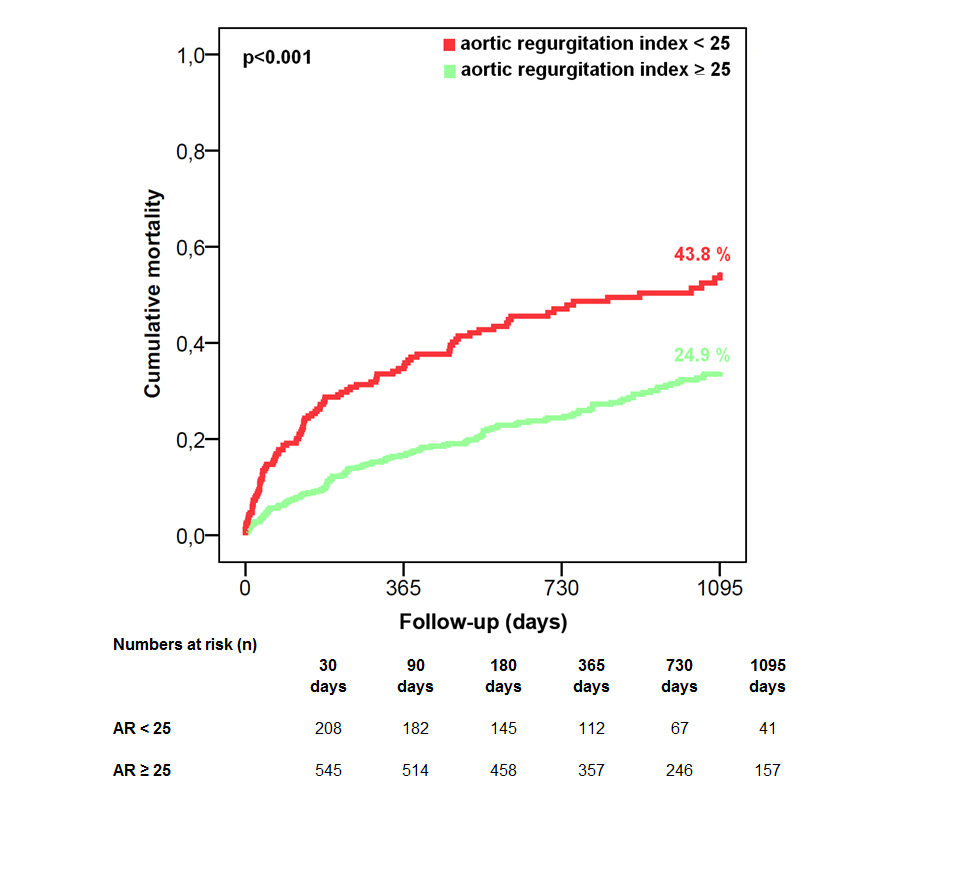

Supplement: S2 Fig — A post-procedural ARI less than 25 was associated with significantly increased all-cause mortality at 3 years (43.8% vs. 24.9%, p<0.001). (TIF) [file pone.0217544.s002.tif]

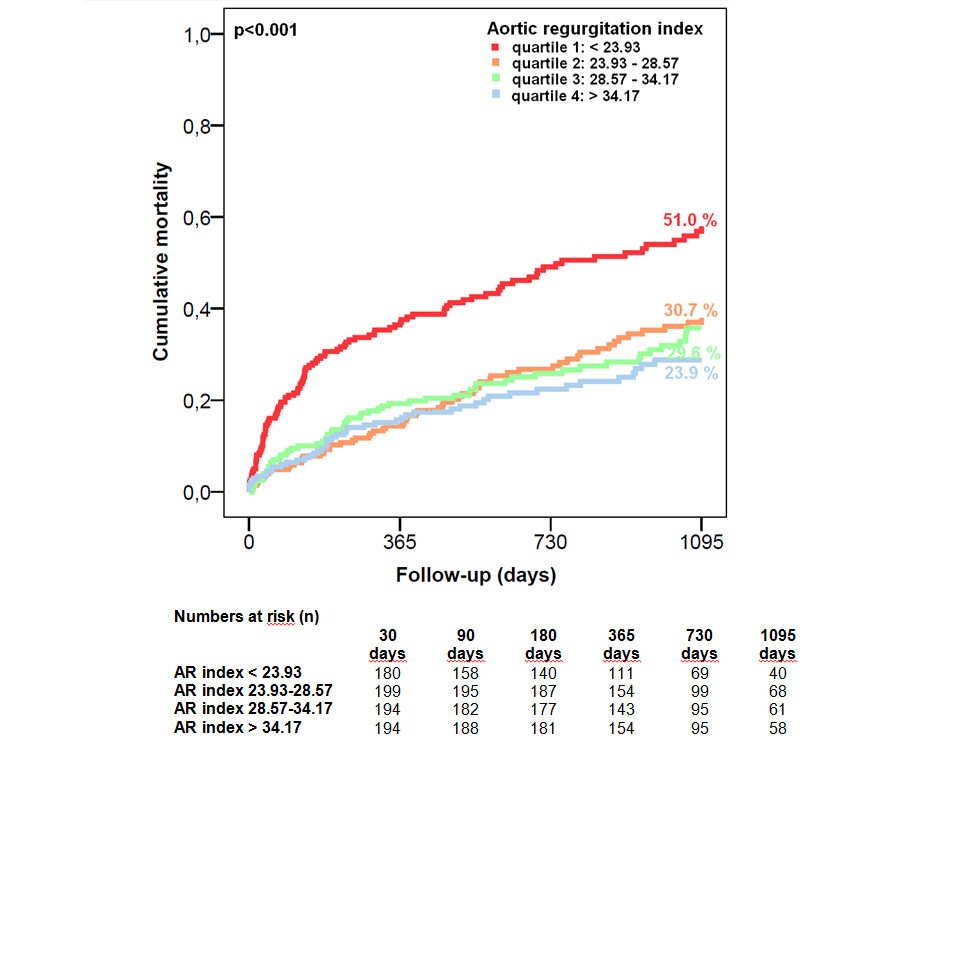

Supplement: S3 Fig — When categorized into quartiles, a post-procedural ARI in the lowermost quartile (<23.93) was significantly associated with increased all-cause mortality at 3 years (Q1: < 23.93: 51.0%, Q2: 23.93–28.57: 30.7%, Q3: 28.57–34.17: 29.6%, Q4: > 34.17: 23.9%; p<0.001). (TIF) [file pone.0217544.s003.tif]

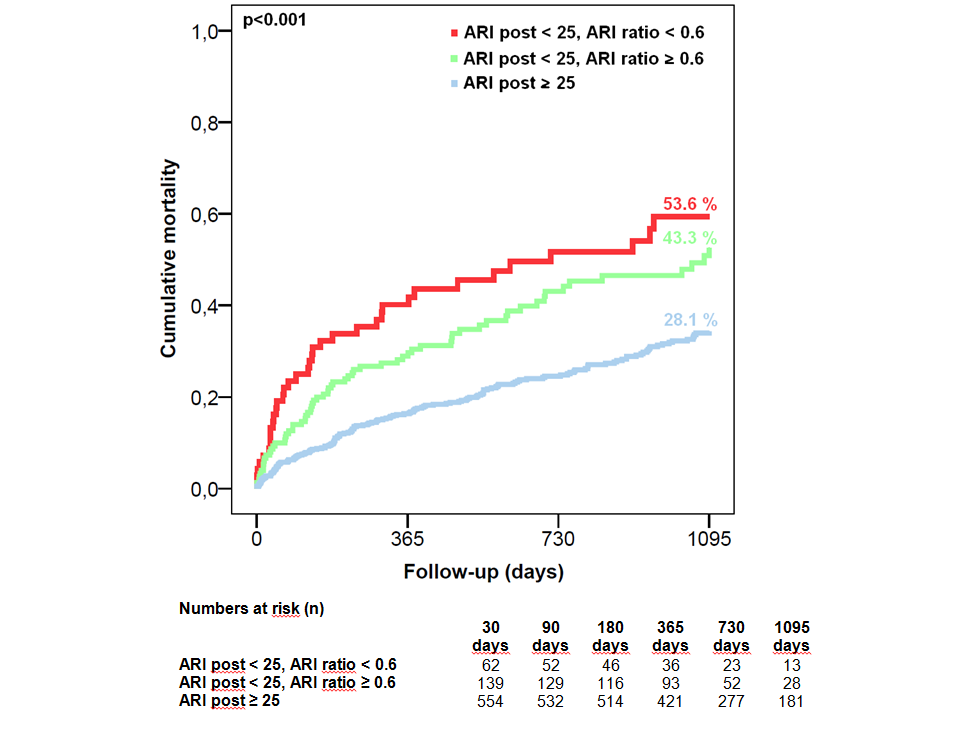

Supplement: S4 Fig — In the entire cohort, TAVI patients with ARIpost < 25 and ARI ratio < 0.60 showed a significant higher 3-year mortality rate (53.6%) compared to those with ARIpost < 25 and ARI ratio ≥ 0.60 (43.3%) or ARIpost ≥ 25 (28.1%), respectively (p<0.001). (TIF) [file pone.0217544.s004.tif]
