# Supplementary material for: Early versus newer generation transcatheter heart valves for transcatheter aortic valve implantation: Echocardiographic and hemodynamic evaluation of an all-comers study cohort using the dimensionless aortic regurgitation index (AR-index)
Source: PLoS One. 2019 May 31;14(5):e0217544. doi: 10.1371/journal.pone.0217544 (PMC6544262; doi:10.1371/journal.pone.0217544)
Supplement: S1 Table — (DOCX) [file pone.0217544.s005.docx]

**Supplemental Table 1 – Baseline characteristics according to transcatheter heart valve type**

|  | **All patients**  **(n=805)** | **Medtronic**  **CoreValve (n=400)** | **Edwards SAPIEN XT (n=48)** | **Direct Flow Medical (n=38)** | **Medtronic**  **Evolut R (n=114)** | **Boston Lotus (n=104)** | **Edwards SAPIEN 3 (n=101)** | **p-value** |
| --- | --- | --- | --- | --- | --- | --- | --- | --- |
|  |  |  |  |  |  |  |  |  |
| Age (years) | **80.9 ± 6.3** | 81.0 ± 6.6 | 81.2 ± 5.1 | 80.9 ± 7.3 | 81.3 ± 5.7 | 80.4 ± 6.6 | 81.0 ± 5.7 | 0.929 |
| Male gender, n (%) | **409 (50.8)** | 210 (52.5) | 24 (50.0) | 28 (73.7) | 32 (28.1) | 52 (50.0) | 63 (62.4) | **<0.001** |
| Logistic EuroSCORE, (%) | **17.5 (11.3 to 29.8)** | 22.6 (13.8 to 36.8) | 12.0 (8.6 to 18.5) | 19.9 (10.6 to 33.3) | 16.9 (11.2 to 24.8) | 12.3 (8.4 to 20.3) | 13.5 (9.4 to 22.2) | **<0.001** |
| EuroSCORE II, (%) | **5.3 (3.3 to 9.4)** | 6.8 (3.9 to 11.5) | 3.5 (2.0 to 7.2) | 4.6 (3.1 to 13.5) | 4.9 (3.0 to 7.6) | 4.6 (3.0 to 7.7) | 4.7 (2.9 to 7.4) | **<0.001** |
| STS-PROM, (%) | **5.2 (3.4 to 8.3)** | 7.0 (4.5 to 10.9) | 5.2 (3.7 to 7.9) | 4.7 (3.2 to 7.7) | 4.2 (2.9 to 5.5) | 3.9 (2.7 to 5.8) | 3.6 (2.3 to 5.1) | **<0.001** |
| Body mass index, (kg/m^2^) | **26.5 ± 5.2** | 26.1 ± 5.4 | 28.2 ± 4.4 | 25.3 ± 3.5 | 27.5 ± 5.4 | 27.0 ± 5.2 | 26.5 ± 5.0 | **0.026** |
| Diabetes mellitus, n (%) | **227 (28.2)** | 112 (28.0) | 17 (35.4) | 13 (34.2) | 29 (25.4) | 27 (26.0) | 29 (28.7) | 0.758 |
| CAD, n (%) | **502 (62.4)** | 263 (65.8) | 28 (58.3) | 27 (71.1) | 59 (51.8) | 60 (57.7) | 65 (64.4) | 0.072 |
| 1-vessel-CAD, n (%) | **168 (20.9)** | 88 (22.0) | 11 (22.9) | 3 (7.9) | 19 (16.7) | 21 (20.2) | 26 (25.7) |  |
| 2-vessel-CAD, n (%) | **129 (16.0)** | 71 (17.8) | 7 (14.6) | 6 (15.8) | 16 (14.0) | 19 (18.3) | 10 (9.9) |  |
| 3-vessel-CAD, n (%) | **206 (25.6)** | 104 (26.0) | 10 (20.8) | 18 (47.4) | 25 (21.9) | 20 (19.2) | 29 (28.7) |  |
| Extracardiac Arteriopathy, n (%) | **344 (42.7)** | 183 (45.8) | 11 (22.9) | 21 (55.3) | 43 (37.7) | 44 (42.3) | 42 (41.6) | **0.025** |
| Atrial fibrillation, n (%) | **338 (42.0)** | 161 (40.3) | 15 (31.3) | 21 (55.3) | 50 (43.9) | 44 (42.3) | 47 (46.5) | 0.257 |
| Previous stroke, n (%) | **122 (15.2)** | 73 (18.3) | 2 (4.2) | 4 (10.5) | 17 (14.9) | 12 (11.5) | 14 (13.9) | 0.097 |
| Previous MI, n (%) | **107 (13.3)** | 78 (19.5) | 2 (4.2) | 5 (13.2) | 8 (7.0) | 7 (6.7) | 7 (6.9) | **<0.001** |
| Previous PCI, n (%) | **289 (35.9)** | 151 (37.8) | 16 (33.3) | 20 (52.6) | 35 (30.7) | 36 (34.6) | 31 (30.7) | 0.159 |
| Previous cardiac surgery, n (%) | **128 (15.9)** | 65 (16.3) | 5 (10.4) | 11 (28.9) | 21 (18.4) | 14 (13.5) | 12 (11.9) | 0.147 |
| COPD, n (%) | **182 (22.6)** | 108 (27.0) | 13 (27.1) | 9 (23.7) | 17 (15.0) | 14 (13.5) | 21 (20.8) | **0.017** |
| Pulmonary hypertension, n (%) | **288 (35.8)** | 142 (35.5) | 8 (16.7) | 13 (34.2) | 48 (42.1) | 36 (34.6) | 41 (41.0) | 0.053 |
| LVEF (%) | **52.6 ± 14.0** | 48.5 ± 15.1 | 57.7 ± 8.9 | 51.5 ± 11.9 | 56.1 ± 12.0 | 57.7 ± 10.6 | 57.4 ± 12.2 | **<0.001** |
| NYHA class IV, n (%) | **100 (12.4)** | 74 (18.5) | 8 (16.7) | 4 (10.5) | 7 (6.1) | 1 (1.0) | 6 (5.9) | **<0.001** |
| Aortic valve area, (cm^2^) | **0.71 ± 0.17** | 0.70 ± 0.17 | 0.74 ± 0.16 | 0.75 ± 0.19 | 0.72 ± 0.18 | 0.73 ± 0.14 | 0.74 ± 0.15 | 0.055 |
| Pressure peak gradient (mmHg) | **73.4 ± 25.9** | 72.5 ± 27.1 | 74.4 ± 24.9 | 69.6 ± 21.9 | 76.5 ± 27.8 | 74.9 ± 23.1 | 73.4 ± 24.1 | 0.710 |
| Pressure mean gradient (mmHg) | **42.0 ± 16.3** | 41.7 ± 17.0 | 41.7 ± 15.3 | 38.8 ± 14.5 | 44.1 ± 18.5 | 43.0 ± 14.5 | 41.1 ± 13.6 | 0.570 |
| CRF, n (%) | **482 (59.9)** | 250 (62.5) | 27 (56.3) | 19 (50.0) | 69 (60.5) | 64 (61.5) | 53 (52.5) | 0.370 |
| eGFR | **52.5 ± 18.1** | 52.1 ± 19.4 | 58.2 ± 22.6 | 54.6± 16.7 | 51.9 ± 15.5 | 51.2 ± 16.0 | 53.0 ± 16.1 | 0.326 |
| Dialysis, n (%) | **28 (3.5)** | 13 (3.3) | 0 (0.0) | 3 (7.9) | 5 (4.4) | 5 (4.8) | 2 (2.0) | 0.358 |
| NT-proBNP (pg/mL) | **2881.0 (1098.5 to 7707.5)** | 3612.0 (1359.5 to 10138.5) | 1901.0 (549.5 to 2938.8) | 2766.0 (1122.5 to 5373.8) | 2345.0 (924.8 to 6095.5) | 2648.0 (725.0 to 5155.0) | 2051.0 (1170.5 to 5363.5) | **<0.001** |
