# Supplementary material for: Early versus newer generation transcatheter heart valves for transcatheter aortic valve implantation: Echocardiographic and hemodynamic evaluation of an all-comers study cohort using the dimensionless aortic regurgitation index (AR-index)
Source: PLoS One. 2019 May 31;14(5):e0217544. doi: 10.1371/journal.pone.0217544 (PMC6544262; doi:10.1371/journal.pone.0217544)
Supplement: S2 Table — (DOCX) [file pone.0217544.s006.docx]

**Supplemental Table 2 – Baseline characteristics according to AR index**

|  | **All patients**  **(n= 805)** | **AR index ≥ 25**  **(n=570)** | **AR index < 25**  **(n=235)** | **p-value** |  |
| --- | --- | --- | --- | --- | --- |
| Age (years) | **80.9 ± 6.3** | 80.7 ± 6.3 | 81.6 ± 6.1 | 0.045 |  |
| Male gender, n (%) | **409 (50.8)** | 290 (50.9) | 119 (50.6) | 0.951 |  |
| Logistic EuroSCORE, (%) | **17.5 (11.3 to 29.8)** | 16.5 (10.4 to 26.6) | 21.1 (12.1 to 35.2) | **< 0.001** |  |
| EuroSCORE II, (%) | **5.3 (3.3 to 9.4)** | 4.9 (3.1 to 9.0) | 6.4 (3.8 to 11.4) | **0.001** |  |
| STS-PROM, (%) | **5.2 (3.4 to 8.3)** | 4.8 (3.2 to 7.8) | 6.1 (3.9 to 10.8) | **< 0.001** |  |
| Body mass index, (kg/m^2^) | **26.5 ± 5.2** | 26.6 ± 5.5 | 26.1 ± 4.4 | 0.217 |  |
| Diabetes mellitus, n (%) | **227 (28.2)** | 150 (26.3) | 77 (32.8) | 0.064 |  |
| CAD, n (%) | **502 (62.4)** | 338 (59.3) | 164 (69.8) | **0.005** | |
| 1-vessel-CAD,  n (%) | **168 (20.9)** | 113 (19.8) | 55 (23.4) |  | |
| 2-vessel-CAD,  n (%) | **129 (16.0)** | 95 (16.7) | 34 (14.5) |  | |
| 3-vessel-CAD,  n (%) | **206 (25.6)** | 131 (23.0) | 75 (31.9) |  | |
| Extracardiac Arteriopathy, n (%) | **344 (42.7)** | 232 (40.7) | 112 (47.7) | 0.070 | |
| Atrial Fibrillation, n (%) | **338 (42.0)** | 250 (43.9) | 88 (37.4) | 0.094 | |
| Previous stroke, n (%) | **122 (15.2)** | 87 (15.3) | 35 (14.9) | 0.894 | |
| Previous MI, n (%) | **107 (13.3)** | 71 (12.5) | 36 (15.3) | 0.277 | |
| Previous PCI, n (%) | **289 (35.9)** | 203 (35.6) | 86 (36.6) | 0.792 | |
| Previous cardiac surgery, n (%) | **128 (15.9)** | 81 (14.2) | 47 (20.0) | **0.041** | |
| COPD, n (%) | **182 (22.6)** | 126 (22.1) | 56 (23.8) | 0.603 | |
| Pulmonary hypertenion, n (%) | **288 (35.8)** | 201 (35.3) | 87 (37.0) | 0.648 | |
| LVEF, (%) | **52.6 ± 14.0** | 52.7 ± 13.6 | 52.3 ± 15.0 | 0.708 | |
| NYHA class IV, n (%) | **100 (12.4)** | 61 (10.7) | 39 (16.6) | **0.021** | |
| Aortic valve area, (cm^2^) | **0.71 ± 0.17** | 0.72 ± 0.17 | 0.71 ± 0.16 | 0.757 | |
| Pressure peak gradient, (mmHg) | **73.4 ± 25.9** | 72.4 ± 24.9 | 75.7 ± 28.2 | 0.115 | |
| Pressure mean gradient, (mmHg) | **42.0 ± 16.3** | 41.2 ± 15.6 | 43.7 ± 17.8 | 0.055 |  |
| CRF, n (%) | **482 (59.9)** | 328 (57.5) | 154 (65.5) | **0.036** |  |
| eGFR | **52.5 ± 18.1** | 53.7 ± 18.1 | 49.6 ± 18.0 | **0.006** |  |
| Dialysis, n (%) | **28 (3.5)** | 10 (1.8) | 18 (7.7) | **<0.001** |  |
| NT-proBNP, (pg/mL) | **2881.0 (1098.5 to 7707.5)** | 2644.5 (942.5 to 6621.5) | 3754.0 (1470.0 to 10588.0) | **< 0.001** |  |
