# Supplementary material for: Early versus newer generation transcatheter heart valves for transcatheter aortic valve implantation: Echocardiographic and hemodynamic evaluation of an all-comers study cohort using the dimensionless aortic regurgitation index (AR-index)
Source: PLoS One. 2019 May 31;14(5):e0217544. doi: 10.1371/journal.pone.0217544 (PMC6544262; doi:10.1371/journal.pone.0217544)
Supplement: S3 Table — (DOCX) [file pone.0217544.s007.docx]

**Supplemental Table 3 – Procedural characteristics according to the transcatheter heart valve type**

|  | **All patients**  **(n=805)** | **Medtronic**  **CoreValve (n=400)** | **Edwards SAPIEN XT (n=48)** | **Direct Flow Medical (n=38)** | **Medtronic**  **Evolut R (n=114)** | **Boston Lotus (n=104)** | **Edwards SAPIEN 3 (n=101)** | **p-value** |
| --- | --- | --- | --- | --- | --- | --- | --- | --- |
|  |  |  |  |  |  |  |  |  |
| Access site |  |  |  |  |  |  |  | **0.016** |
| Trans-femoral, n (%) | **785 (97.5)** | 380 (95.0) | 48 (100.0) | 38 (100.0) | 114 (100.0) | 104 (100.0) | 101 (100.0) |  |
| Trans-subclavian, n (%) | **14 (1.7)** | 14 (3.5) | 0 (0.0) | 0 (0.0) | 0 (0.0) | 0 (0.0) | 0 (0.0) |  |
| Trans-apical, n (%) | **0 (0.0)** | 0 (0.0) | 0 (0.0) | 0 (0.0) | 0 (0.0) | 0 (0.0) | 0 (0.0) |  |
| Trans-aortic, n (%) | **6 (0.7)** | 6 (1.5) | 0 (0.0) | 0 (0.0) | 0 (0.0) | 0 (0.0) | 0 (0.0) |  |
| Prosthesis size |  |  |  |  |  |  |  | **<0.001** |
| 20 mm, n (%) | **1 (0.1)** | 0 (0.0) | 1 (2.1) | 0 (0.0) | 0 (0.0) | 0 (0.0) | 0 (0.0) |  |
| 23 mm, n (%) | **96 (11.9)** | 17 (4.3) | 15 (31.3) | 2 (5.3) | 15 (13.2) | 24 (23.1) | 23 (22.8) |  |
| 25 mm, n (%) | **49 (6.1)** | 0 (0.0) | 0 (0.0) | 11 (28.9) | 0 (0.0) | 38 (36.5) | 0 (0.0) |  |
| 26 mm, n (%) | **230 (28.6)** | 134 (33.5) | 27 (56.3) | 0 (0.0) | 39 (34.2) | 0 (0.0) | 30 (29.7) |  |
| 27 mm, n (%) | **54 (6.7)** | 0 (0.0) | 0 (0.0) | 12 (31.6) | 0 (0.0) | 42 (40.4) | 0 (0.0) |  |
| 29 mm, n (%) | **308 (38.3)** | 182 (45.5) | 5 (10.4) | 13 (34.2) | 60 (52.6) | 0 (0.0) | 48 (47.5) |  |
| 31 mm, n (%) | **67 (8.3)** | 67 (16.8) | 0 (0.0) | 0 (0.0) | 0 (0.0) | 0 (0.0) | 0 (0.0) |  |
| Annulus diameter, (mm | **23.9 ± 2.4** | 23.6 ± 2.4 | 23.2 ± 2.0 | 25.0 ± 2.8 | 23.1 ± 2.1 | 23.9 ± 1.7 | 25.5 ± 2.4 | **<0.001** |
| Maximum diameter, (mm) | **26.8 ± 2.8** | 26.4± 2.8 | 26.0 ± 2.3 | 27.5 ± 2.8 | 26.0 ± 2.5 | 27.6 ± 1.8 | 29.4 ± 2.5 | **<0.001** |
| Minimum diameter, (mm) | **21.0 ± 2.4** | 20.6 ± 2.2 | 20.6 ± 1.9 | 21.2 ± 2.4 | 20.4 ± 2.7 | 21.3 ± 1.7 | 23.2 ± 2.5 | **<0.001** |
| Pre-procedural aortic regurgitation index (AR index) | **34.2 ± 10.5** | 33.3 ± 10.6 | 33.2 ± 9.9 | 36.2 ± 10.7 | 32.8 ± 10.7 | 36.5 ± 11.2 | 35.9 ± 8.9 | **0.020** |
| Pre-dilation, n (%) | **408 (50.7)** | 211 (52.8) | 45 (93.8) | 38 (100.0) | 34 (29.8) | 12 (11.5) | 68 (68.0) | **<0.001** |
| Post-dilation, n (%) | **200 (24.8)** | 146 (36.5) | 9 (18.8) | 1 (2.6) | 37 (32.5) | 4 (3.8) | 3 (3.0) | **<0.001** |
| Procedure time (min.) | **63.0 (50.0 to 86.0)** | 65.5 (50.0 to 89.0) | 68.0 (55.5 to 85.0) | 77.5 (57.8 to 96.3) | 57.5 (47.8 to 79.0) | 62.0 (52.0 to 83.5) | 53.5 (44.0 to 77.3) | **0.001** |
