# Supplementary material for: Early versus newer generation transcatheter heart valves for transcatheter aortic valve implantation: Echocardiographic and hemodynamic evaluation of an all-comers study cohort using the dimensionless aortic regurgitation index (AR-index)
Source: PLoS One. 2019 May 31;14(5):e0217544. doi: 10.1371/journal.pone.0217544 (PMC6544262; doi:10.1371/journal.pone.0217544)
Supplement: S4 Table — (DOCX) [file pone.0217544.s008.docx]

| **Supplemental Table 4 – Clinical and functional outcomes according to the transcatheter heart valve type** | | | | | | | | |
| --- | --- | --- | --- | --- | --- | --- | --- | --- |
|  | **All patients**  **(n=805)** | **Medtronic**  **CoreValve (n=400)** | **Edwards SAPIEN XT (n=48)** | **Direct Flow Medical (n=38)** | **Medtronic**  **Evolut R (n=114)** | **Boston Lotus (n=104)** | **Edwards SAPIEN 3 (n=101)** | **p-value** |
|  |  |  |  |  |  |  |  |  |
| 30-day mortality, n (%) | **39 (4.8)** | 30 (7.5) | 0 (0.0) | 0 (0.0) | 2 (1.8) | 2 (1.9) | 5 (5.0) | **0.011** |
| 180-day mortality, n (%) | **116 (14.4)** | 82 (20.5) | 3 (6.3) | 3 (7.9) | 9 (7.9) | 7 (6.7) | 12 (11.9) | **<0.001** |
| 1-year mortality, n (%) | **162 (20.1)** | 114 (28.5) | 8 (16.7) | 5 (13.2) | 14 (12.3) | 8 (7.7) | 13 (12.9) | **<0.001** |
| 2-year mortality, n (%) | **211 (26.2)** | 147 (36.8) | 15 (31.3) | 9 (23.7) | 14 (12..3) | 10 (9.6) | 16 (15.8) | **<0.001** |
| 3-year mortality, n (%) | **245 (30.4)** | 173 (43.3) | 20 (41.7) | 10 (26.3) | 14 (12.3) | 10 (9.6) | 18 (17.8) | **<0.001** |
| Stroke, n (%) | **20 (2.5)** | 14 (3.5) | 0 (0.0) | 0 (0.0) | 0 (0.0) | 4 (3.8) | 2 (2.0) | 0.174 |
| Myocardial infarction, n (%) | **6 (0.7)** | 5 (1.3) | 0 (0.0) | 1 (2.6) | 0 (0.0) | 0 (0.0) | 0 (0.0) | 0.310 |
| Minor vascular complications, n (%) | **154 (19.1)** | 81 (20.3) | 15 (31.3) | 6 (15.8) | 15 (13.2) | 22 (21.2) | 15 (14.9) | 0.099 |
| Major vascular complications, n (%) | **32 (4.0)** | 26 (6.5) | 1 (2.1) | 0 (0.0) | 1 (0.9) | 1 (1.0) | 3 (3.0) | **0.014** |
| Major bleedings, n (%) | **36 (4.5)** | 32 (8.0) | 1 (2.1) | 0 (0.0) | 0 (0.0) | 1 (1.0) | 2 (2.0) | **<0.001** |
| Pacemaker implantation, n (%) | **124 (15.4)** | 73 (18.3) | 2 (4.2) | 3 (7.9) | 16 (14.0) | 24 (23.1) | 6 (5.9) | **0.009** |
| Acute kidney injury, n (%) | **125 (15.5)** | 86 (21.5) | 7 (14.6) | 6 (15.8) | 8 (7.0) | 13 (12.5) | 5 (5.0) | **<0.001** |
| Angiographic data |  |  |  |  |  |  |  | **<0.001** |
| None pAR, n (%) | **342 (42.5)** | 113 (28.2) | 13 (27.1) | 18 (47.4) | 46 (40.4) | 92 (88.5) | 60 (59.4) |  |
| Mild pAR, n (%) | **403 (50.1)** | 241 (60.3) | 29 (60.4) | 17 (44.7) | 63 (55.3) | 12 (11.5) | 41 (40.6) |  |
| Moderate pAR, n (%) | **55 (6.8)** | 41 (10.3) | 6 (12.5) | 3 (7.9) | 5 (4.4) | 0 (0.0) | 0 (0.0) |  |
| Severe pAR, n (%) | **5 (0.6)** | 5 (1.3) | 0 (0.0) | 0 (0.0) | 0 (0.0) | 0 (0.0) | 0 (0.0) |  |
| More than mild pAR, n (%) | **59 (7.3)** | 45 (11.3) | 6 (12.5) | 2 (5.3) | 6 (5.3) | 0 (0.0) | 0(0.0) | **<0.001** |
| Post-procedural aortic regurgitation index (AR index, ARI) | **29.2 ± 8.4** | 28.1 ± 8.3 | 27.7 ± 7.2 | 30.8 ± 6.9 | 29.6 ± 9.2 | 34.2 ± 8.7 | 28.4 ± 6.9 | **<0.001** |
| AR index < 25, n (%) | **235 (29.2)** | 133 (33.3) | 14 (29.2) | 6 (15.8) | 36 (31.6) | 14 (13.5) | 32 (31.7) | **0.002** |
| ARI ratio | 0.92 ± 0.43 | 0.93 ± 0.53 | 0.88 ± 0.26 | 0.92 ± 0.35 | 0.97 ± 0.39 | 1.00 ± 0.35 | 0.82 ± 0.19 | 0.076 |
