# Supplementary material for: Early versus newer generation transcatheter heart valves for transcatheter aortic valve implantation: Echocardiographic and hemodynamic evaluation of an all-comers study cohort using the dimensionless aortic regurgitation index (AR-index)
Source: PLoS One. 2019 May 31;14(5):e0217544. doi: 10.1371/journal.pone.0217544 (PMC6544262; doi:10.1371/journal.pone.0217544)
Supplement: S5 Table — (DOCX) [file pone.0217544.s009.docx]

**Supplemental Table 5 – Severity of pAR and AR index**

|  | **AR index ≥ 25** | **AR index < 25** | **p-value** |
| --- | --- | --- | --- |
| Paravalvular aortic regurgitation | | | **<0.001** |
| None | 263 (46.1%) | 63 (26.8%) |  |
| Mild | 293 (51.4%) | 127 (54.0%) |  |
| Moderate | 14 (2.5 %) | 40 (17.0%) |  |
| Severe | 0 (0.0 %) | 5 (2.1%) |  |
